# Effect of respiratory muscle training on functional capacity related to the quality of life of patients undergoing coronary artery bypass grafting with cardiopulmonary bypass: randomized clinical trial

**DOI:** 10.31744/einstein_journal/2026AO1720

**Published:** 2026-01-02

**Authors:** Mary Silva da Cruz Neves Ribeiro, Fabiana Della Via, Antônio Luís Eiras Falcão, Antonio Francisco de Oliveira, Carolina Kosour

**Affiliations:** 1 Universidade Federal de Alfenas Alfenas MG Brazil Universidade Federal de Alfenas, Alfenas, MG, Brazil.; 2 Universidade Estadual de Campinas Campinas SP Brazil Universidade Estadual de Campinas, Campinas, SP, Brazil.

**Keywords:** Thoracic surgery, Breathing exercises, Rehabilitation, Physical therapy modalities

## Abstract

**Objective:**

To evaluate the effects of inspiratory muscle training using POWERbreathe® in patients undergoing myocardial revascularization with extracorporeal circulation, focusing on respiratory and peripheral muscle strength, exercise capacity, functional independence, and quality of life, from hospitalization to a 30 and 90 day followup.

**Methods:**

This randomized controlled clinical trial included 70 patients aged 44-78 years who were randomized into two groups. The Control Group underwent conventional physical therapy (Control Group, n=34), whereas the Intervention Group underwent conventional physical therapy combined with inspiratory muscle training using POWERbreathe® (Intervention Group, n=36). Respiratory and peripheral muscle strength, capacity, functional independence, and quality of life were evaluated preoperatively and 5, 30, and 90 days after surgery.

**Results:**

A statistically significant increase in exercise capacity in the Intervention Group was observed 90 days after surgery (p=0.0192), as well as in the functional capacity domain of quality of life at 30 and 90 days after surgery (p=0.0079). The maximal inspiratory pressure and handgrip strength showed a negative correlation, ranging from weak to moderate intensity, in both groups over time.

**Conclusion:**

Inspiratory muscle training with the POWERBreathe combined with physical therapy improves exercise capacity and quality of life in patients undergoing myocardial revascularization.

**Prospero database registration:** RBR-2p4mz5.

## Highlights

■Cardiovascular diseases reduce exercise tolerance and impair quality of life.

■Quality of life monitoring is essential after cardiac surgery.

■Poor postoperative quality of life predicts worse functional recovery.

■Inspiratory muscle training improves exercise capacity and quality of life.

## Summary

Patients undergoing coronary artery bypass grafting with cardiopulmonary bypass frequently experience reduced functional capacity, respiratory impairment, and decreased quality of life. Despite advances in perioperative care, evidence of the role of inspiratory muscle training in this setting is limited. Ribeiro et al. proposed a randomized controlled clinical trial to evaluate the effects of inspiratory muscle training using an electronic device (POWERbreathe®) in patients undergoing cardiac surgery with cardiopulmonary bypass.

## Graphical abstract

**Figure:**
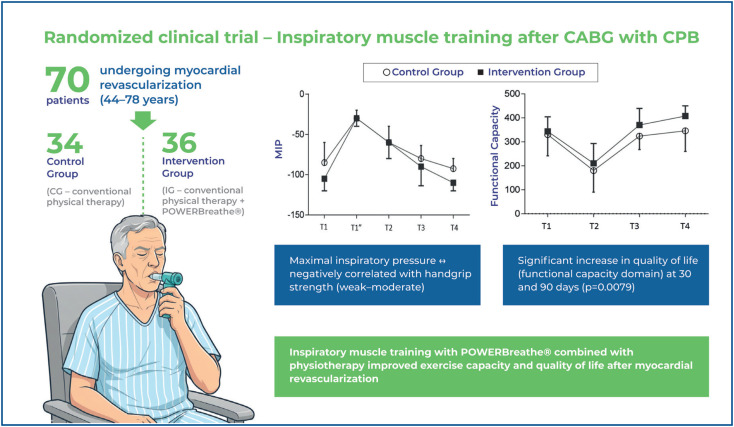

## INTRODUCTION

Cardiorespiratory and musculoskeletal deconditioning may occur following coronary artery bypass graft surgery.^(1)^ Thus, patients who undergo coronary artery bypass graft surgery frequently experience reduced exercise capacity^(2)^ and diminished functional independence^(3)^ which negatively affect their quality of life.^(4)^

Additionally, surgical procedures, including anesthesia and mechanical ventilation, can impair pulmonary mechanics and gas exchange.^(5)^ These ventilatory dysfunctions become even more pronounced with the use of cardiopulmonary bypass (CPB), which is associated with the release of inflammatory mediators triggered by aortic clamping and reperfusion injury,^(6)^ ultimately leading to respiratory muscle dysfunction.^(2,6)^

Quality of life, particularly its physical dimension, is a key factor that must be monitored after cardiac surgery, as it is directly related to recovery. Poor postoperative quality of life predicts poor recovery in terms of functional status. In this regard, studies have demonstrated that respiratory muscle training and aerobic exercise can mitigate the decline in respiratory muscle strength and enhance exercise capacity in these patients.^(2)^ Although inspiratory muscle training (IMT) using electronic devices has proven to be effective in cardiovascular rehabilitation,^(7)^ its use in postoperative settings remains understudied.

## OBJECTIVE

This study aimed to evaluate the effects of inspiratory muscle training using the POWERbreathe® device in patients undergoing myocardial revascularization with cardiopulmonary bypass, focusing on respiratory and peripheral muscle strength, exercise capacity, functional independence, and quality of life during hospitalization and at 30- and 90-day follow-ups.

## METHODS

### Study design

This randomized controlled clinical trial was approved by the Research Ethics Committee of the *Universidade Federal de Alfenas* (UNIFAL) under Protocol CAAE: 68758017.2.0000.5142; #2.195.884, has been registered in the Brazilian Registry of Clinical Trials (RBR-2p4mz5). All participating patients provided informed consent by signing an Informed Consent Form. The original study design was retained throughout the study. Due to the nature of the intervention (electronic device use with visual feedback), blinding of the participants and physiotherapists was not feasible.

### Participants

This study enrolled adult patients aged >18 years who required elective coronary artery bypass grafting (CABG) with CPB at our hospital. The inclusion criteria were as follows: 1) presence of cardiovascular instability in the pre-or postoperative period; 2) cardiac operation associated with myocardial revascularization; and 3) operative reintervention. Patients with neurological, cognitive, or musculoskeletal impairment were excluded from the study.

### Randomization

Eligible patients were randomized into the Control Group (CG) and the Intervention Group (IG). After patient selection, randomization was performed on an electronic platform (www.randomization.com) using a numerical list with a randomized sequence of simple allocations.

### Interventions

The CG included patients who underwent conventional physical therapy starting on the immediate postoperative day (POI) until hospital discharge. APhysiotherapy sessions were conducted according to the protocol described in Table 1S, Supplementary Material. Both protocols were performed by researchers.

Patients in the IG underwent the same conventional physical therapy protocol as those in the CG; however, the protocol was performed using an electronic device (POWERBreathe® International Ltd, Warwickshire, UK, Ironman K5 model) specifically designed for respiratory muscle training. The electronic device was programmed to be in automatic mode with a light intensity equivalent to 40% of the maximum value obtained in the first inspiration performed on the device. The adjustment at each session, as well as load grading, was performed automatically using Breathe-Link software (Version 1.0, 2012). Starting from the first day after the operation (1st PO), patients were positioned in the armchair, and three sets of 10 inspirations were performed in the mouthpiece of the electronic device, using a nose clip, twice a day, until the fifth day after the operation (5th PO), during the period of hospitalization. The POWERbreathe device used in this study was approved by the Brazilian Health Regulatory Agency (ANVISA - *Agência Nacional de Vigilância Sanitária*), registration number 81001390001).

During the follow-up periods of 30 and 90 days after the operation, all patients were instructed to proceed with home training according to the conventional physiotherapy protocol (identical for both groups), previously trained and made available in printed material for the activities to continue. The protocol involved exercises on the fifth day after surgery and an increase in the time of daily walking, with an initial fractional duration of 10 min weekly, progressing until reaching 25 min, according to the patient's functionality.

The patients included in this study did not receive additional therapy or guidance from other physical therapists following the guidelines of this protocol.

### Recorded assessments and times

We reviewed the patients’ electronic medical records and conducted interviews to collect data on basic patient characteristics, type and date of operation, medications, and comorbidities.

Respiratory muscle strength and exercise capacity were defined as the primary study outcomes, whereas quality of life after surgery was the secondary outcome. Patient assessments included maximal inspiratory pressure (MIP) and maximal expiratory pressure measurements, six-minute walk test (6 MWT), Functional Independence Measure (FIM), handgrip strength test, global muscle strength test, and quality of life using the Medical Outcomes Study 36 - Item Short-Form Health Survey - SF-36" (SF-36). A single examiner performed the assessments in the hospital ward, before and after the intervention period in five stages: T1: pre-operative, on the day before the operation; T1": 30 min after the spontaneous breathing test, before extubation, in the Intensive Care Unit (ICU); T2: five days after the operation; T3, 30 days after the operation; and T4, 90 days after the operation. Additionally, pulmonary measurements were used to assess respiratory performance in the IG, including tidal volume, flow, pressure, strength, and energy of the inspiratory muscles, according to data provided by the Breathe-Link software in the electronic device.

### Respiratory muscle strength

We assessed respiratory muscle strength according to the recommendation of the Guideline for Pulmonary Function Tests, of the Brazilian Society of Pneumology and Tisiology.^(8)^ Specifically, we measured MIP and maximal expiratory pressure, using an analog manovacuometer (Comercial Médica®, model M120), with an operational range of −120 to +120 cmH_2_O, attached to a mouthpiece, using a nose clip, in the sitting position at 90o. The patients were instructed to perform maximal expiration up to the residual volume, followed by maximal inspiration to measure MIP.^(9)^ Similarly, the patients performed maximum and slow inspirations until they reached total lung capacity, followed by forced maximal expiration for the maximal expiratory pressure assessment. The measured pressures were sustained for at least 1 s, in a total of three maneuvers, with an interval of 1 min between them. The maneuver with the highest value was considered for analysis.^(10)^ A standardized verbal stimulus was provided for maneuvering performance.^(9)^ If the patient was in the ICU during the period prior to extubation, the position adopted during assessment supine at 45o by occlusion of the manovacuometer in the endotracheal tube. This position was maintained for 30 s in the three measurements, with a 1 min interval between them, without using a one-way valve. Respiratory muscle strength was assessed at T1, T1", T2, T3, and T4.

### Exercise capacity

We assessed tolerance to exercise capacity using the 6 MWT, according to the American Thoracic Society Guidelines^(11)^ in a continuous and flat 30-m corridor. Patients were instructed to walk as quickly as possible without running. They were allowed to slow down, stop, rest if necessary, and resume walking as quickly as possible. During the journey, the examiner accompanied the patient, positioning himself posterolaterally to continuously monitor heart rate, peripheral oxygen saturation, and patient safety, in addition to performing standardized verbal stimulation.^(12)^ The patients underwent two tests: a learning test, and an assessment test performed after 1h using the highest score for analysis.^(11)^ Exercise capacity was assessed at times T1, T2, T3, and T4.

### Functional Independence Assessment

Functional independence was assessed using the FIM scale, a multidimensional instrument that measures the degree of difficulty or limitation attributed to each volunteer. The FIM consists of 18 items classified into six dimensions and two subdivisions: motor and cognitive. Each item has a score of seven levels to assess the need for help in performing activities of daily living, with possible scores ranging from 18-126. The higher the score, the higher the level of independence and functional performance.^(13)^ Functional independence was assessed at times T1, T2, T3 and T4.

### Peripheral and global muscle strength

Peripheral muscle strength was determined using a handgrip strength test with a hydraulic dynamometer (Saehan Corporation, model SH5001, 630-728, Korea). The participants were instructed to remain seated in a chair without arms, with the spine erect, maintaining the knee flexion angle at 90°, the shoulder positioned in adduction and neutral rotation, the elbow flexed at 90°, forearm semi-pronation, wrist neutral, and the arm suspended in the air. The hand was positioned on the dynamometer and adjusted to the second position according to the recommendations of the American Society of Hand Therapists.^(14)^ Three measurements were taken with the dominant hand for 3 s of maximum contraction, with a 1-min rest between each measurement. The largest measure was used for analysis. A standardized verbal stimulus was used for measurement.^(15)^

We assessed global muscle strength based on the Medical Research Council (MRC) scale, grading muscle strength from 0 (absence of contraction) to 5 (normal muscle strength) in the main groups and identifying the degree of weakness by the abduction movements of the shoulder, elbow flexion, wrist extension, hip flexion, knee extension, and ankle dorsiflexion. The total score ranges from zero (complete tetraparesis) to 60 (normal muscle strength).^(16)^

### Quality of life

We assessed quality of life according to the Medical Outcomes Study 36 - Item Short-Form Health Survey - SF-36" (SF-36), translated and adapted to Portuguese.^(17)^ This multidimensional instrument consists of 36 items grouped into eight domains: functional capacity, physical aspects, pain, general health, vitality, social aspects, emotional aspects, and mental health. The final score ranges from 0-100, corresponding to the worst and best general health statuses, respectively.^(17)^

### Intraoperative and immediate postoperative procedure

Anesthesia was induced intravenously with fentanyl, midazolam, etomidate, and pancuronium, and general anesthesia was maintained using isoflurane inhalation.

After the surgical procedure, the patients were transferred to an ICU unit adapted to a microprocessed ventilator (DIXTAL®, model 3010), with pressure-controlled ventilation with an 80% inspired oxygen fraction (FiO_2_), tidal volume of 6ml/kg /predicted weight, respiratory rate of 12 rpm, positive end-expiratory pressure - Positive End Expiratory Pressure (PEEP) of 6 cmH_2_O.

The preserved capacity to initiate inspiratory efforts, adequate level of consciousness (Glasgow Coma Scale >8), acid-base balance, partial pressure of oxygen in the arterial blood (PaO_2_) ≥60 mmHg, and inspired fraction of oxygen (FiO_2_) ≤0.4 and PEEP ≤5 to 8 cmH_2_O,^(18)^ were the ventilatory parameters for pressure support ventilation that were changed for patients with hemodynamic stability and the ventilatory weaning process was started.

We performed a spontaneous breathing test using a T-tube coupled to a Venturi system with 40% FiO_2_ for 30-40 min. Subsequently, the patients who were considered fit were extubated and maintained on oxygen therapy through a Venturi mask, 40% FiO_2_. Arterial blood gases were collected to determine the degree of impairment in pulmonary gas exchange, oxygenation indices, and alveolar-arterial oxygen differences before anesthetic induction, 1 h after surgery, 30 min after the spontaneous breathing test, before extubation and 6 h after surgery.

### Statistical analysis

The required sample size was calculated based on the results of a previous study, considering a confidence level of 95% and a margin of error of 5%, with a sample size of 46 individuals.^(19)^

We used the Mann-Whitney, chi-square, Fisher's exact test, and comparisons by deltas, based on the Mann-Whitney test to compare the numerical and categorical variables in the preoperative period. The Friedman and Dunn tests were used to compare the numerical variables of the electronic device. Analysis of variance for repeated measures with rank transformation was employed to analyze the groups and times, followed by a contrast profile test.

Additionally, we used rank transformation due to the variability of the measures and the absence of a normal distribution, which was assessed using the Shapiro-Wilk test. We also tested the effects of group, time, and their interaction. In the presence of a significant interaction, its breakdown was used to analyze the effects of each time point on the groups. In contrast, each effect was independently assessed in the absence of a significant interaction.

Correlation analysis was based on Spearman's correlation coefficients. Intention-to-treat treatments were not administered. All statistical analysis were performed using the Statistical Analysis System, 9.4 (SAS Institute Inc., Cary, NC, USA), with statistical significance set at p<0.05. Groups were masked to perform data analysis.

## RESULTS

**Figure 1:**
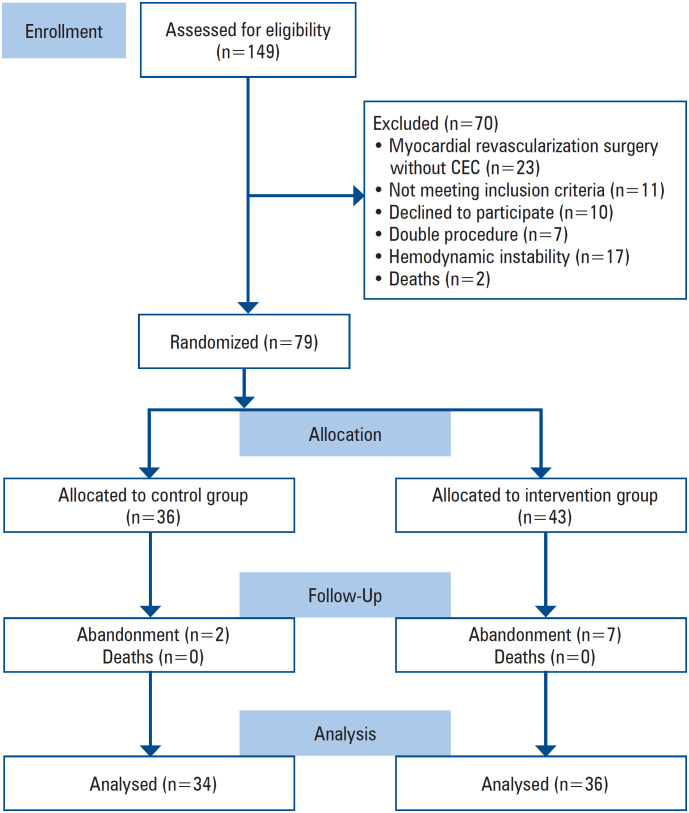
Patient selection process

Of the 149 enrolled patients, 79 met the inclusion criteria. Figure 1 shows the study flowchart according to the Consolidated Standards of Reporting Trials (CONSORT).

The data in table 1 demonstrate the methodological rigor applied to the clinical and operative variables to allow the groups to be compared without the influence of pre- and/or intraoperative factors. No statistically significant differences were observed between the groups in terms of demographic data, comorbidities, or operative risk, as well as in the immediate intra- and postoperative periods and ICU stay (Table 1).

**Table 1:** Demographic, surgical and postoperative data, comorbidities, and intensive care unit and hospital lengths of stay

Variables		Control Group (n=34)	Intervention Group (n=36)	p value
Age (years)		65.5 ± 7.4	62 ± 7.7	0.0605†
Sex				
	Female	9	7	0.4841†
	Male	25	29	
Body mass index (Kg/m^2^)		27.1 ± 3.5	27.1 ± 3.1	0.7025†
Hypertension (n/%)				
	No	10 (29)	10 (28)	0.8798◊
	Yes	24 (71)	26 (72)	
*Diabetes mellitus* (n/%)				
	No	15 (44)	23 (64)	0.0970◊
	Yes	19 (56)	13 (36)	
Dyslipidemia (n/%)				
	No	14 (41)	16 (44)	0.7824◊
	Yes	20 (59)	20 (56)	
Smoking (n/%)				
	Neves smoked	10 (29)	10 (28)	
	Former smoker	17 (50)	17 (47)	0.9080◊
	Smoker	7 (21)	9 (25)	
Family history of coronary artery disease (n/%)				
	No	15 (44)	15 (42)	0.8359◊
	Yes	19 (56)	21 (58)	
Medical diagnostic				
	Unstable angina	17 (50)	24 (67)	
	Acute non-ST segment elevation myocardial infarction	6 (18)	5 (14)	0.3465◊
	Acute myocardial infarction with ST segment elevation	11 (32)	7 (19)	
Injury severity				
	Single vessel	0 (0)	1 (3)	
	Two-vessel	3 (9)	7 (20)	0.3266ϕ
	Three-vessel	26 (76)	23 (66)	
	Left main coronary artery lesion	5 (15)	4 (11)	
Operating time (min)		3.6 ± 0.4	3.83 ± 0.4	0.0687
CEC time (min)		63.9 ± 11.6	66 ± 14.4	0.5409
Aortic clamping (min)		41.6 ± 9.5	43.8 ± 11.5	0.4412
Mechanical ventilation time (h)		6.7 ± 2.8	5.7 ± 2.5	0.1023
Grafts per patient (n)		2.9 ± 0.6	2.8 ± 0.9	0.4472
Arterial grafts (n)		1.1 ± 0.2	1.1 ± 0.2	0.9650
Vein grafts (n)		1.8 ± 0.8	1.7 ± 0.9	0.4216
Body temperature (ºC)		33.5 ± 0.6	33.6 ± 0.4	0.5813
Bleending in drain −12 h (mL)		483 ± 272.9	472.6 ± 156.3	0.4706
Bleending in drain - 24 h (mL)		720 ± 363.7	716.3 ± 194.3	0.4294
Fluid balance intraoperative		1434.7 ± 532.6	1356.9 ± 693.6	0.4099
Fluid balance in 12 h (mL)		930.8 ± 942.1	835.9 ± 739.8	0.7971
Fluid balance in 24 hs (mL)		1427.5 ± 1074.3	1235.7 ± 858.8	0.5984
APACHE II		11.3 ± 4.1	9.4 ± 2.5	0.3298
SOFA		2.9 ± 1.4	2.6 ± 1.1	0.1064
Length of stay in the ICU (d)		2 ± 1.1	1.8 ± 0.9	0.4022
Length of stay in the hospital (d)		8.7 ± 2.4	9 ± 4	0.8569

† Mann–Whitney test;

◊ Chi-square test;

ϕ Fisher's exact test. p<0.05.

EuroSCORE II: European System for Cardiac Operative Risk Evaluation; ASA: American Society of Anesthesiologists; CEC: extracorporeal circulation; ICU: intensive care unit; APACHE II: Acute Physiology and Chronic Health Evaluation; SOFA: sequential organ failure assessment.

Similarly, no significant differences were observed between the groups regarding the administration of blood products, red blood cell concentrates, platelets, and fresh frozen plasma used intraoperatively and postoperatively in the first 24 h, as well as pulmonary gas exchange and ventilatory mechanics data during the operation (p>0.05).

### Muscle strength and functionality

IMT resulted in a longer distance walked during the 6-min walk test at T4, which showed a statistically significant interaction between groups and times (p=0.0251) and a difference between groups (p=0.0192). The delta analysis generated a similar result (p=0.0414) for the IG (Table 2).

**Table 2:** Respiratory, peripheral and global muscle strength, exercise capacity and functional independence over time

Variables		Time			Variance analysis				
		T1	T1"	T2	T3	T4	Interaction GxT	Group	p value
Maximal inspiratory pressure (cmH_2_O)									
	CG	-85.4 ± 28	-31.7 ±14†	-59 ± 25† ϕ	-82.1 ± 27.3† ϕ ◊	-90.6 ± 25.7† ϕ ◊ #	0.8821	0.5831	<0.0001
	IG	-91.3 ± 33.1	-32.5 ± 13.3†	-61.9 ± 24.6† ϕ	-84.4 ± 30.8† ϕ ◊	-95.3 ± 29.7† ϕ ◊ #			
Maximal expiratory pressure (cmH_2_O)									
	CG	96 ± 30.4	22.9 ± 13†	79.2 ± 29.5† ϕ	103.8 ± 25.7ϕ ◊	107.8 ± 23.2† ϕ ◊ #	0.8441	0.7174	<0.0001
	IG	99.3 ± 31.8	22.8 ± 9.8†	78.3 ± 25.9† ϕ	104.5 ± 23.7ϕ ◊	108.1 ± 22.6† ϕ ◊ #			
Handgrip strength (Kg)									
	CG	29.3 ± 8.9	9.4 ±8.6†	25.9 ± 8.4† ϕ	28.8 ± 8.7ϕ ◊	30.6 ± 9.1ϕ ◊ #	0.5506	0.0557	<0.0001
	IG	33 ± 9.9	13.3 ± 8.7†	30.4 ± 8.8† ϕ	32.3 ± 9.1ϕ ◊	34 ± 8.7 ϕ ◊ #			
Global muscle strength									
	CG	59.3 ± 1.5	35 ± 10.7†	55 ± 4.2† ϕ	58.6 ± 2.2ϕ ◊	57.6 ± 9.5† ϕ ◊ #	0.2393	0.0821	<0.0001
	IG	59.4 ± 1.3	35.3 ± 10.7†	57 ± 2.5† ϕ	59.2 ± 1.4ϕ ◊	59.8 ± 0.7† ϕ ◊ #			
Functional Independence									
	CG	124 ± 4.7	-	100.4 ± 9.18†	118.1 ±10.1† ϕ	123.2 ± 5.1◊ #	0.5345	0.2043	0.0001
	IG	124 ± 5.9	-	102.1 ± 8.5†	121.7 ±6.3† ◊	124.5 ± 3.4◊ #			
Exercise capacity (m)									
	CG	313.7 ± 99.5	-	205 ± 121.2†	330.3 ± 94.5◊	340.6 ± 102.1◊	0.0251	0.1765	<0.0001
	IG	325.1 ± 95.7	-	217 ± 82.1†	363.3 ± 89.7† ◊	397.2 ± 85.5† ◊ # ϕ			

† Compared to the T1;

ϕ Compared to the T1;

◊ Compared to the T2;

# Compared to the T3;

ϕ interaction group-time; p<0.05.

T1: pre-operative; T1": 30 min after the spontaneous breathing test; T2,5 days after the operation; T3, 30 days after the operation; T4, 90 days after the operation; G, group.

The effect of time was statistically significant for IG, showing an increase in the distance covered over the analyzed times (T1-T3, T1-T4, T2-T3, T2-T4, and T3-T4). Meanwhile, the CG only showed a significant increase in the distance covered in times T2-T3 and T2-T4.

The delta analyses showed no statistically significant differences or statistically significant interactions between groups and times for respiratory, peripheral, and global muscle strength and the measure of functional independence. Only the effect of time showed a significant difference between both groups for respiratory, peripheral, and global muscle strength and the measure of functional independence.

The correlation between MIP and handgrip strength, global muscle strength, and exercise capacity at T1 revealed a weak-to-moderate negative correlation in the total cohort. At T3 and T4, both groups presented a weak to moderate negative correlation between MIP, handgrip strength, and functional capacity.

### Ventilation parameters

Pulmonary measurements (tidal volume, flow, pressure, strength, and energy of inspiratory muscles) for the IG provided by the Breathe-Link software during the five days of IMT were significantly higher in the 3rd, 4th, and 5th sessions than that in the first session. The absence of a significant difference between the first and second sessions can be explained by the reduction in chest mobility caused by the presence of pleural and mediastinal drains that were removed on postoperative day two. Consequently, a gradual improvement in all variables occurred from the 2nd session onwards compared with the previous day's session during the five sessions of IMT (Table 3).

**Table 3:** Data from the electronic device throughout the first to fifth session of respiratory muscle training in the intervention group

Variable	D1	D 2	D 3	D 4	D 5	D 5 - D 1 (%)	p value
Volume (mL)	0.57±0.28	0.55±0.23	0.69±0.24† ϕ	0.74±0.35† ϕ	0.84±0.37† ϕ ◊ #	64.49±78.31	<0.0001
Flow (L/s)	0.68±0.45	0.68±0.31	0.97±0.52† ϕ	1.08±0.54† ϕ	1.25±0.62† ϕ ◊	104.77±84.66	<0.0001
Pressure (cmH_2_O)	7.41±3.17	8.37±3.35	10.33±4.3† ϕ	11.16±4.22† ϕ	13.03±5.44† ϕ ◊ #	84.83±80.21	<0.0001
Strenght (watts)	0.63±0.81	0.68±0.62†	1.20±1.11† ϕ	1.41±1.08† ϕ ◊	1.92±1.72† ϕ ◊ #	297.19±299.16	<0.0001
Energy (joule)	11.83±13.29	13.29±6.51	22.89±14.66† ϕ	26.23±17.1† ϕ ◊	34.11±20.75† ϕ ◊ #	226.55±272.28	<0.0001

† compared to session 1;

ϕ compared to session 2;

◊ compared to session 3;

# compared to session 4; p<0,05.

D1: average of two sessions on the first day; D2: average of two sessions on the second day; D3: average of two sessions on the third day; D4: average of two sessions on the fourth day; D5: average of two sessions on the fifth day; D5-D1: percentage difference between the average of the fifth day and the first day.

### Quality of life

Quality of life measured by the functional capacity domain of the SF-36 instrument showed a statistically significant interaction between groups and times (p=0.0079) and a significant difference between groups, in which the IG had higher values at times T3-T4 than the CG (Table 4). Therefore, we performed an interaction breakdown between groups and times for the functional capacity domain. Individual analysis of time effects on the groups revealed no significant differences between the groups at T1 and T2. In contrast, a significant difference was observed for T3 and T4 between the groups (p=0.0444 and p=0.0353, respectively), with the IG presenting higher values in the functional capacity domain than the CG.

**Table 4:** Quality of life domains obtained over the periods studied

Variable		Time				Variance analysis		
		T1	T2	T3	T4	Interaction GxT	Group	p value
Functional capacity								
	CG	62.5± 24	26.3 ±17#	50.6 ± 21.3# ϕ ◊	64 ± 23.7ϕ ◊	0.0079	0.1514	< 0.0001
	IG	55 ± 25.1	32.6 ± 15#	61.0 ± 23.1† ϕ ◊ #	75.3±18.6† # ϕ ◊ ▲			
Physical aspect								
	CG	25.7 ± 38.7	9.6 ± 23.8† #	9.6 ± 20.4ϕ	27.2 ± 33.4# ϕ ◊	0.0679	0.4288	0.0001
	IG	9.7 ± 19.2	0.7 ± 4.2#	8.3 ± 14.6ϕ	34.7 ± 35# ϕ ◊			
Pain								
	CG	65.8 ± 25.6	49.1 ± 24.8#	66 ± 20.1ϕ	75.3 ± 20.1# ϕ ◊	0.3019	0.0966	< 0.0001
	IG	65.6 ± 28.9	60.9 ±28.8#	74.9 ± 21.3ϕ	78.9 ± 18.1# ϕ ◊			
General health								
	CG	80.2 ± 15.8	84.2 ± 14.2#	87 ±10.6#	88.1 ± 9.9#	0.6631	0.3592	0.0082
	IG	79.9 ±16.2	82.4 ±16.8#	82.6 ± 13.5#	85.7 ± 11.2#			
Vitality								
	CG	68.1 ± 24.1	65.4 ± 29.4	76.8 ± 18.4# ϕ	76.9± 20.2# ϕ	0.9381	0.6631	0.0001
	IG	66.3 ± 27.5	68.7 ± 23.2	78.2 ± 17.8# ϕ	80 ± 17.6# ϕ			
Social aspects								
	CG	66.3 ± 31.1	51.8 ± 31.9#	60.6 ± 22.3ϕ	78.3 ± 21.4# ϕ ◊	0.7350	0.3268	0.0001
	IG	69.2 ± 29.1	56.6 ± 27.6#	70.6 ± 23.4ϕ	80 ± 25.3# ϕ ◊			
Emotional aspects								
	CG	38.1 ± 40.3	30.4 ± 37.1	41.1 ± 35ϕ	59.6 ± 39.9# ϕ ◊	0.9019	0.5289	0.0001
	IG	33.3 ± 37.4	24.9 ± 33.2	37 ± 40.2ϕ	59.2 ± 36.7# ϕ ◊			
Mental health								
	CG	72.4 ± 27.2	77.8 ± 22.8	82.5 ± 14.3# ϕ	84.4 ± 14.3# ϕ ◊	0.6487	0.2658	0.0006
	IG	67.2 ± 25	70.9 ± 25.6	80.4 ± 18# ϕ	79.2 ± 20.1# ϕ ◊			

† Comparison between groups, Mann–Whitney test;

# compared to T1;

ϕ compared to T2;

◊ compared to T3;

▲ interaction group-time; p<0,05.

Abbreviations: T1, preoperative; T2,5 days after the operation; T3, 30 days after the operation; T4, 90 days after the operation.

The Mann-Whitney U test further confirmed these results for the domain of functional capacity of quality of life, with the IG showing a significant difference (T3, p=0.0360; T4, p=0, 0450). Additionally, the delta analysis for the functional capacity domain of quality of life indicated a significant difference in the IG at all times compared to T1 (T2, p=0.0230; T3, p=0.0153; T4, p=0.0049), as well as in the domain of limitation due to the physical aspects of T4 (p=0.0052). The remaining domains-pain, general health, vitality, social aspects, emotional aspects, and mental health-showed no significant differences in the interaction between groups and times or in the analysis between the groups.

## DISCUSSION

The results of this randomized clinical trial demonstrated that compared to physical therapy alone, a combination of physical theraoy and IMT improved exercise capacity and quality of life in patients during the postoperative period after myocardial revascularization.

At the five-day reassessment after surgery, a reduction in inspiratory muscle strength was observed in both groups, followed by a gradual increase throughout the follow-up period, with no significant differences between the groups, which is consistent with previous studies.^(20)^ According to previous studoes, IMT increases MIP and improves inspiratory muscle strength when the values are below 70% of the predicted value.^(21)^ However, in this study, respiratory muscle training at a low intensity (40% of MIP) over a short intervention period did not significantly change the MIP, although it led to improvements in functional capacity. This result may be attributed to the type of intervention used, which involved automatic load adjustment based on individual patient needs, unlike previous studies that used fixed loads, which likely provided more efficient respiratory muscle activation.^(22)^

Pulmonary metrics obtained from the device software showed progressive and significant improvements over the five training sessions, which is consistent with the findings of Romanini et al., despite no significant changes in MIP^(23)^ These improvements may be related to reduced ventilatory functional limitations in the postoperative period, which may have facilitated increased mobility, decreased respiratory muscle weakness, and improved neuromuscular mechanics.

Notably, these ventilatory metrics demonstrated progressive gains over each training session, suggesting that such parameters may serve as an alternative and potentially more sensitive approach for monitoring respiratory capacity and strength, as opposed to relying solely on MIP values.^(24)^ Additionally, the IG demonstrated superior performance in the 6-min walk test, compared to that observed in the CG, which is consistent with the results of recent studies.^(24)^ As suggested by Zanini et al.,(20) physiological adaptations induced by physical training may alleviate pain in the saphenous vein donor limb, thereby improving the performance on functional tests. These factors likely contributed to the improved exercise performance when combined with the enhanced oxygen delivery promoted by IMT. The observed enhancement in exercise capacity may also be partially explained by attenuation of the inspiratory muscle metaboreflex. IMT increases the aerobic capacity of inspiratory muscles, reduces anaerobic metabolism, delays the onset of respiratory muscle fatigue, and increases blood flow to the peripheral muscles, thereby improving physical performance.^(5,24)^

Another noteworthy finding of this study was the inverse correlation between the MIP and handgrip strength with weak-to-moderate intensity. This negative correlation may reflect compensatory mechanisms during postoperative recovery, such as redistribution of functional muscle demand between the respiratory and peripheral systems. Although initially counterintuitive, this finding does not contradict the overall benefits of IMT, but underscores the complexity of neuromuscular interactions during recovery and highlights the need for future studies to further explore how regional muscle strength adapts to systemic stress. Notably, improvements in functional capacity and quality of life were observed despite this inverse association, indicating that IMT contributes positively to patient outcomes, even when some physiological metrics may not improve in parallel.

Quality of life, particularly in the functional capacity domain, significantly improved in the IG compared to that in the CG, corroborating the results of a recent meta-analysis.^(25)^ In contrast, the CG showed slower recovery in this domain at T3 and a significant decline when compared to baseline (T1), which was not observed in the IG. The functional capacity domain is directly related to patients’ physical performance and may benefit from the reduced negative impact of the inspiratory metaboreflex, leading to improved exercise tolerance.^(25)^

Despite the statistically significant improvements in exercise capacity and quality of life, their clinical relevance should be interpreted with caution considering the modest sample size and effect magnitude. Additionally, the follow-up period revealed persistent limitations in domains related to physical and emotional aspects, even at 90 days after surgery. These findings emphasize the importance of long-term multidisciplinary follow-up during the recovery process of patients undergoing cardiac surgery.

## Limitations

Despite the positive outcomes, this study has some limitations. First, the study had a relatively small sample size and short duration of IMT. Second, the observed effect sizes were modest and may raise concerns regarding clinical feasibility, particularly when considering the costs associated with the use of electronic devices. Nevertheless, the pulmonary parameters obtained through the device provided detailed insights into the patients’ real-time functional recovery. These data offer valuable contributions to future studies exploring respiratory capacity and strength more comprehensively, particularly regarding the limitations of using MIP as a standalone indicator of respiratory muscle performance. Further research employing tools such as electroneuromyography and ultrasound may help better elucidate these mechanisms and guide more targeted interventions for respiratory muscle dysfunction.

## CONCLUSION

This randomized controlled trial contributes to the growing body of scientific evidence supporting the use of inspiratory muscle training in physical therapy with the aim of enhancing the functional capacity and quality of life in patients undergoing coronary artery bypass grafting with cardiopulmonary bypass.

## Data Availability

The underlying content is contained within the manuscript.
