# Supplementary material for: Effect of respiratory muscle training on functional capacity related to the quality of life of patients undergoing coronary artery bypass grafting with cardiopulmonary bypass: randomized clinical trial
Source: Einstein (Sao Paulo). 2026 Jan 2;24:eAO1720. doi: 10.31744/einstein_journal/2026AO1720 (PMC12977242; doi:10.31744/einstein_journal/2026AO1720)
Supplement: SUPPLEMENTARY MATERIAL [file 2317-6385-eins-24-eAO1720-suppl1.pdf]

SUPPLEMENTARY MATERIAL

Effect of respiratory muscle training on functional capacity related to the quality of life of patients undergoing coronary artery bypass grafting with cardiopulmonary bypass: Randomized clinical trial

Mary Silva da Cruz Neves Ribeiro, Fabiana Della Via, Antônio Luís Eiras Falcão, Antonio Francisco de Oliveira Neto, Carolina Kosour

DOI: 10.31744/einstein_journal/2026AO1720

Table 1S. Conventional physiotherapy protocol exercises immediate postoperative period

Patient in Fowler's position
Weaning from mechanical ventilation
1st Postoperative Day
Patients are seated in a chair
1. Diaphragmatic breathing exercises
2. Wrist flexion and extension exercises
3. Knee extension exercises
4. Ankle dorsiflexion and plantar flexion exercises
2 sets of 10 repetitions for each exercise, twice a day
2nd Postoperative Day
Patients are seated in a chair
1. Diaphragmatic breathing exercises
2. Wrist flexion and extension exercises
3. Shoulder flexion and extension exercises associated with deep inspiration
4. Knee extension exercises
5. Ankle dorsiflexion and plantar flexion exercises
2 sets of 10 repetitions for each exercise, twice a day
3rd Postoperative Day
Patients are seated in a chair
1. Incentive spirometry
2. Assisted walking for 35 meters
3. Exercises from the previous day, with progression to 3 sets of 10 repetitions for each exercise, twice a day
4th Postoperative Day
Patients are seated in a chair
1. Assisted walking for 100 meters
2. Exercises from the previous day
3 sets of 10 repetitions for each exercise, twice a day
5th Postoperative Day
Patients are seated in a chair
1. Assisted walking for 165 meters
2. Exercises from the previous day
3 sets of 10 repetitions for each exercise, twice a day
